# Childhood obesity prevention in schools and municipalities – the digital planning tool WEPI

**DOI:** 10.1093/eurpub/ckac131.425

**Published:** 2022-10-25

**Authors:** M Schröder, R Berner, B Babitsch, H Hassel

**Affiliations:** 1 Institute for Applied Health Sciences, Coburg University of Applied Sciences and Art, Coburg, Germany; 2 Department of New Public Health, Osnabrück University, Osnabrück, Germany

## Abstract

**Background:**

The development of theory-based prevention efforts is complex and often not feasible in the daily work of project planners. Planning tools such as the Intervention Mapping Approach (IMA), which is a gold standard for project planning, cannot be used in practice without preconditions. How can prevention planning be evidence-based but also practical? Against this background, the research project WEPI was developed, funded by the Federal Ministry of Health, Germany. WEPI is a digital planning tool based on the IMA, that guides municipalities and schools in planning childhood obesity prevention efforts.

**Methods:**

From April 2019 to October 2020, the planning tool was developed. In October 2020 and February 2021, WEPI was tested for the first time by selected municipalities (n = 4) in Bavaria and schools (n = 4) in Lower Saxony. Based on this, the modified planning tool was tested for a second time throughout Germany (05-11/2021). Handling and acceptance were evaluated in a questionnaire survey.

**Results:**

The first test showed that content and technical aspects needed to be optimised in order to bring scientific demands and practical feasibility together. Six municipalities and eight educational institutions participated in the second test phase. The respondents (n = 14) indicated that WEPI facilitates structured project planning (86%) and supports collaboration with colleagues (64%). 93% would use the planning tool again. WEPI offers evidence-based methods for practical implementation as well as a download area, including a template for a project application or a project summary. This service was also evaluated as very helpful (64%).

**Conclusions:**

Through step-by-step guidance, WEPI facilitates evidence-based project planning and ensures planning quality. Effort and benefit are in good proportion. Further optimisations are needed to improve the user-friendliness.

**Key messages:**

• WEPI provides a comprehensive roadmap, helping practitioners to approach a structured project planning.

• Practitioners’ participation promotes acceptance and provides a user-friendly development.

